# Dyadic relationship, carer role, and resources: a theory-driven thematic analysis of interviews with informal carers focusing on the stability of home-based care arrangements for people living with dementia

**DOI:** 10.1186/s12877-022-03618-y

**Published:** 2022-11-28

**Authors:** Kerstin Köhler, Jan Dreyer, Iris Hochgraeber, Christiane Pinkert, Milena von Kutzleben, Bernhard Holle, Martina Roes

**Affiliations:** 1 Deutsches Zentrum Für Neurodegenerative Erkrankungen (DZNE), Witten, Germany; 2 Department of Nursing Science, Witten/Herdecke University, Faculty of Health, Witten, Germany; 3 Department of Health Services Research, Carl Von Ossietzky Universität Oldenburg, Faculty of Medicine and Health Sciences, Oldenburg, Germany

**Keywords:** Dementia, Home-based care, Informal care, Caregiver, Care arrangement, Stability, Thematic qualitative text analysis

## Abstract

**Background:**

Most people with dementia live at home and are supported by informal carers. During the care trajectory, the creation of a stable care situation is a guiding principle of informal carers, who often manage complex care arrangements. A recently developed theory – ‘Stability of home-based care arrangements for people living with dementia' (SoCA-Dem) – conceptualises how such care arrangements develop over time, and it highlights the relevance of the dyadic relationship, carer role, and resources with regard to the continuation of home-based care throughout the course of dementia. To further explore these three concepts, and to provide feedback for a further refinement of SoCA-Dem theory, this study aims to gain a deeper understanding of (1) how informal carers perceive their dyadic relationship, their carer role, and the resources of the care arrangement, and (2) how these concepts are interrelated.

**Methods:**

This study was a secondary data analysis of *n* = 11 problem-centred interviews. Data were interpreted by a thematic qualitative text analysis.

**Results:**

The findings distinguished subthemes within the concepts and uncovered their interrelations. The kinship relation, living situation and character of the dyadic relationship shaped informal carers’ self-conception of the carer role. This influenced the integration of resources into the care arrangement. Conversely, the quantity and quality of informal and formal support resulted in a feeling of relief or overload in the carer role, that shaped the informal carers’ way of living their dyadic relationship. The respective forming of the concepts had a significant impact with regard to the perceived stability or instability of the care situation in the examined care arrangements.

**Conclusions:**

This study provided valuable evidence for future research alignment and targeted refinement of the SoCA-Dem theory. Scholars should further explore the specifics of spousal versus parent–child-dyads to better understand the dyads' diverse strategies in the creation of stable home-based care arrangements. Furthermore, future research should focus on the complex dynamics of dyads, family networks, and service providers, and all actors’ perspectives should be integrated in SoCA-Dem theory. Based on this growing knowledge base, innovative care interventions and structures should be developed to support people with dementia and their informal carers in better living and caring in the place of their choice.

## Background

Worldwide, most people with dementia live at home [1] and express the explicit wish to stay in their familiar environments for as long as possible [2]. Driven by the motivation to support the person living with dementia in the best sense possible [3], informal carers are a vital resource and a cornerstone for community-based dementia care [4].

Usually, informal carers live in a close, long-standing relationship to the person with dementia, which is exposed to change but continues over the trajectory [5, 6]. Informal carers provide emotional support and practical supervision and care, serve as gatekeepers between the informal and formal support systems, and manage care arrangements that are often complex [7, 8]. Most informal carers undergo periods of burden, but they also report positive experiences throughout the care trajectory [9, 10].

Due to the progressive nature of dementia, informal carers are challenged to adjust to the ever-changing care situation [11] while they seek to ‘keep things in balance’ [12]. In this process, their overarching goal is to create and maintain a stable care situation [13]. However, while some carers succeed in making arrangements that maintain stability over years, others struggle with the challenges they face, fail to master crises and initiate the move to an institutional care setting quite early in the trajectory [14, 15].

Since contemporary socio-political programs such as ‘Ageing in Place’ [16] have declared ageing in one’s own home as their targeted goal, there is a growing body of scientific knowledge regarding home-based care in the context of dementia. As informal carers are central players that enable community-based dementia care many studies explore the multifaceted experience of informal carers, for example their understanding and forming of the carer role [17, 18], their needs [19–21], their burden [9, 22, 23], their positive experiences [4, 10], their strategies to make decisions [24, 25] and to constantly adapt care [12, 13, 26], and many other aspects. Other studies highlight the interaction between diverse actors involved in home-based dementia care, for example interaction within dyads [3, 6, 27, 28] and family networks [29–31], as well as between family carers and professionals [32–34]. But in most of these studies the researched experiences and interactions are not directly linked to whether they contribute to a stable care situation at home or not. In addition, there is plenty of research on interventions that aim to promote stable home-based care arrangements and to delay nursing home admissions. Recent meta-analyses [35, 36] suggest that only complex, multicomponent interventions contributed to prolong care at home. Thus, many facets of home-based dementia care are well researched, but the complex interplay of these diverse facets and how this interplay affects the stability of home-based care arrangements, still needs further investigation.

**Fig. 1:**
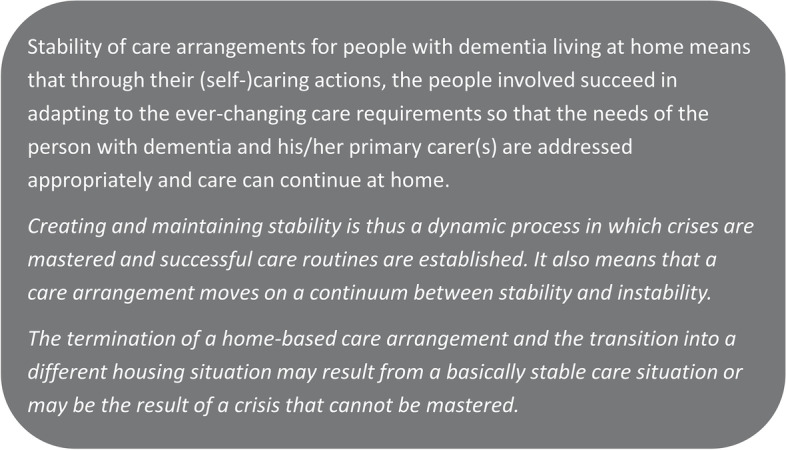
Definition of stability of home-based care arrangements for people living with dementia [37, 38]

The significance of the interaction between the person with dementia, the informal carer, and the informal and formal support network – while striving to maintain care at home over time – is highlighted in a working definition of ‘stability of home-based care arrangements’ [37] (Fig. 1). In this definition, stability is conceptualised as a dynamic process that unfolds over the trajectory of dementia care, that is intentionally shaped by the actors, and that can be achieved only if the needs of the person with dementia and the informal carer are addressed.

**Fig. 2:**
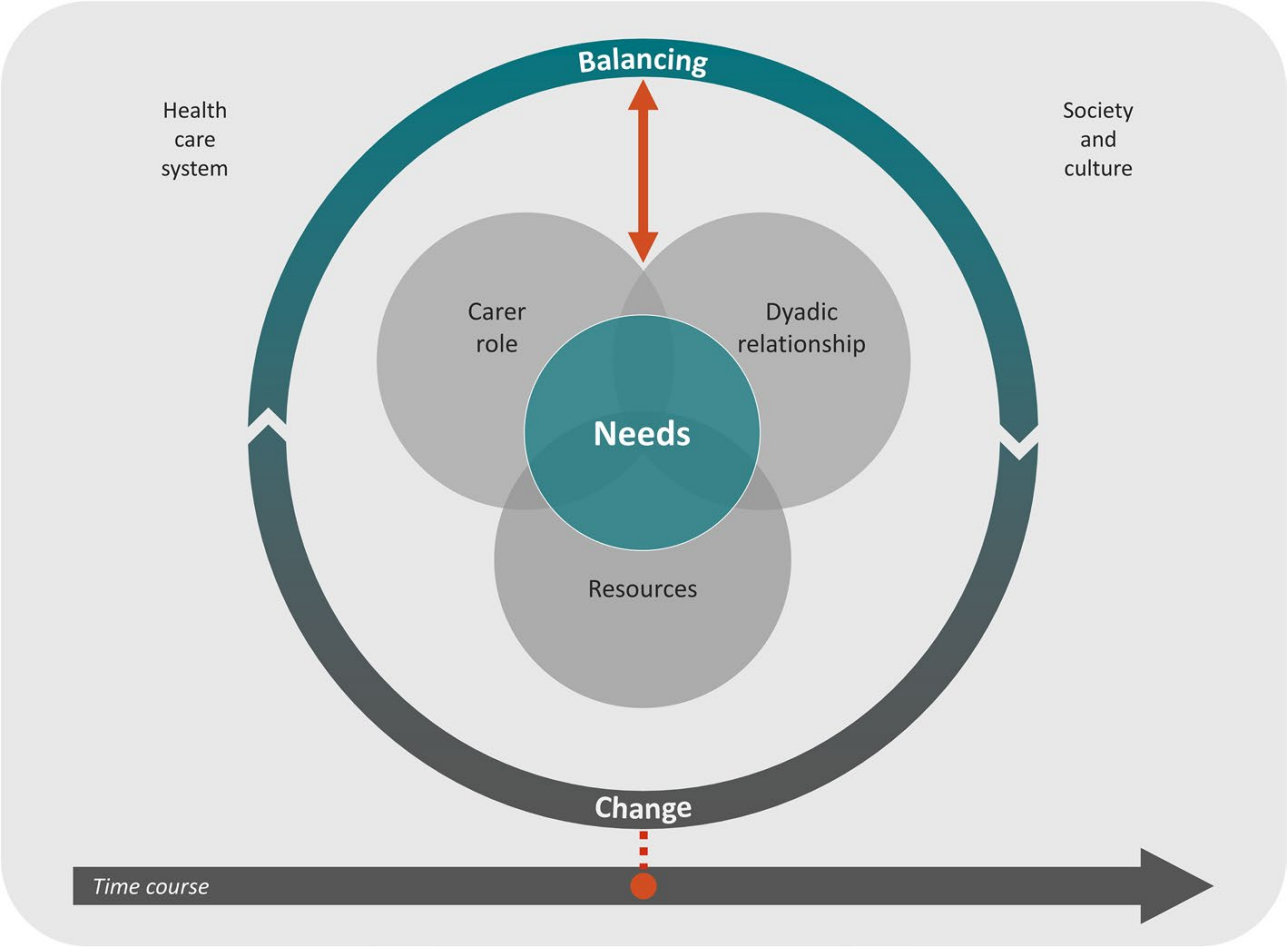
Conceptual model of stability of home-based care arrangements for people living with dementia [38]

This study is part of the ‘Stability of home-based care arrangements’ (SoCA) research project of the Deutsches Zentrum für Neurodegenerative Erkrankungen (DZNE), site Witten. Within this project, we explore the complex phenomenon of stability from theory to intervention. Based on the cited definition [37], as a first step in the SoCA project we developed a middle-range theory of ‘stability of home-based care arrangements for people living with dementia’ (SoCA-Dem) through a meta-study [38]. This theory serves as the theoretical frame for the current study, and it conceptualises the phenomenon of stability from the perspective of informal carers. In the context of the SoCA project, we defined an informal carer as a relative, friend or neighbour who provides ongoing assistance to the person living with dementia, and who is usually unpaid. A conceptual model of SoCA-Dem theory (Fig. 2) visualises the key concepts that together constitute the stability of a home-based care arrangement.

From the perspective of the informal carer, at any time, *change* may occur and trigger an action (*balancing*) of the informal carer. How the informal carer balances the change influences whether the *needs* of the person with dementia and the informal carer are met. The balancing shapes – and in turn is shaped by – the *dyadic relationship*, the informal carer’s experience of the *carer role*, and the management of *resources*. All of this is embedded in the context of the specific *society and culture* and *health care system*.

The character and temporal development of the *dyadic relationship*, *carer role* and *resources* either promote a stable care situation or provoke unstable phases. SoCA-Dem theory indicates a dynamic interplay of these three concepts, but details of how they are entwined remain rather vague.

Additional empirical research is needed to overcome this vagueness of the recently developed middle-range theory.

Middle-range theories are intermediate in their level of abstraction between unifying grand theories and working hypotheses of day-to-day research [39, 40]. They are explicitly meant to guide empirical research [41, 42] and can, therefore, be moved forward in their explanatory power [43]. Smith and colleagues noted that there is ‘continued work necessary to grow ideas over time’ [44]. This is the intention of the SoCA-Dem project. Following the theory-building meta-study, we now use the SoCA-Dem theory in empirical studies with a broad spectrum of methodologies. For example, a quantitative study that developed a typology of care arrangements has been recently published [45]. This study is the first qualitative study to utilise the SoCA-Dem theory as its theoretical basis. It aims to deepen the understanding of selected theory concepts and to provide feedback for a further refinement of the SoCA-Dem theory.

## Methods

### Aim and research questions

The aim of this study is to gain a deeper understanding of the *dyadic relationship*, *carer role*, and *resources* of home-based dementia care arrangements and to further clarify the overlap between these concepts that were deduced from the SoCA-Dem theory. The following research questions guided the analysis:

- How do informal carers of a person living with dementia describe and perceive their dyadic relationship, their carer role, and the resources of their care arrangement?
- How are these three concepts interrelated?

### Study design

This is a qualitative secondary data analysis (SDA) of *n* = 11 problem-centred interviews [46] with informal carers of a person living with dementia. This study is part of the SoCA (Stability of Home-based Care Arrangements) project line at the Deutsches Zentrum für Neurodegenerative Erkrankungen (DZNE), site Witten. The reporting of this publication is in accordance with the Standards for Reporting Qualitative Research (SRQR) [47] and with the recommendations on conducting SDA provided by Ruggiano and Perry [48]. The primary study that delivered the data used aimed primarily to validate a phase model of informal dementia care trajectories [13] and to provide a first collection of the perceptions of informal carers with regard to the phenomenon of stability. In this SDA, we focus on stability-related topics that were considered to be at the fringe in the primary study. Most of the authors (K.K., J.D., I.H., M.v.K., and B.H.) were involved in the design and data collection of the primary study and were familiar with its context, which helped to minimise the chances of a misinterpretation of the original data.

### Recruiting and data collection

Participants for the primary study were recruited via Alzheimer’s societies and welfare organisations, especially in day care centres and self-help groups for informal carers, as well as via practice partners who were involved in ongoing research projects at the DZNE site Witten. The recruiting procedure was a purposive sampling strategy targeting the creation of a heterogeneous sample according to the sociodemographic characteristics of the informal carers and the people living with dementia. Oral and written information was provided to the interested informal carers, and their written informed consent was obtained. The recruited participants chose whether the interview should take place in their private home, in a public place, or via telephone. K.K., J.D. and M.v.K. performed the problem-centred interviews (which lasted between 49 and 94 min) between July 2016 and May 2017. The problem-centred interview is an interview technique that enables the researcher to overcome the alleged contradiction between being directed by theory or being open-minded such that an interplay of inductive and deductive thinking contributes to the generation of further knowledge [46]. We strived for a high proportion of narration and asked few additional direct questions. The interviews were recorded and transcribed verbatim by a professional transcription service. In addition, each interviewee completed a short questionnaire to report relevant sociodemographic data. All data were pseudonymised before analysis. All of the participants agreed with the usage of data in future secondary analyses at the DZNE.

### Subsample selection

For the primary study, *n* = 3 focus group interviews, *n* = 2 double interviews (one couple and two informal carers), and *n* = 11 single interviews were conducted. For this SDA, we selected the subsample of the *n* = 11 single interviews. The rationale for this selection was that in this SDA, we aimed to explore the individual care experience of single informal carers (within-case perspective) and to subsequently analyse similarities and differences across the individual care arrangements (cross-case perspective). This publication focuses on the reporting of the cross-case results related to the targeted concepts.

### Data analysis

The data analysis was performed primarily by the first author (K.K.) but in continuous feedback loops with the whole author team.

To become familiar with the data, K.K. initially listened to the audio recordings of the interviews, carefully read the transcripts and wrote a preliminary case summary for each of the *n* = 11 care arrangements. To perform the data analysis, we used MAXQDA 2020 software. The interviews were analysed using thematic qualitative text analysis [49], a method that allows for the deduction of thematic categories from a theory (as we intended) and is suitable for SDA.

An initial coding scheme, including the concepts of *dyadic relationship*, *carer role*, and *resources* as thematic categories, was deduced from SoCA-Dem theory. In the first coding round, the initial coding scheme was complemented by the inductive creation of further themes and subthemes related to the concepts. Definitions for each theme and subtheme were elaborated and applied in a second coding round using the extended coding scheme. In a consensual coding process [50], *n* = 4 interviews were coded by the first author and an additional co-author/colleague (J.D., C.P., B.H., or J.I.B.). Discrepancies in the coding were discussed until consensus was reached, and the coding scheme was then further refined. After the consensual coding, the final coding scheme was determined, and all *n* = 11 interviews were coded again.

In the next step, a thematic matrix [49] was generated by using the ‘summary grid’ functions of MAXQDA 2020. For each interview (within-case), we created analytical summaries of the coding assigned to each subtheme and compiled them into summaries of the main concepts, *dyadic relationship, carer role*, and *resources*. Afterwards, we wrote cross-case summaries for each subtheme and each main concept. The process of matrix generation and the content of the matrix were discussed among the author team on a regular basis.

Based on the coding process and the thematic matrix and in close iterative discourses with the whole author team, KK interpreted the informal carers’ statements regarding the concepts *carer role*, *dyadic relationship* and *resources*; the relationships between the themes and subthemes of one main concept; and the relationships between the concepts and the themes and subthemes. This was done by checking the within-case and cross-case summaries for references to other concepts and subthemes and by structuring these references. The interpretation phase was supported by the visual tool MAXMaps of MAXQDA 2020. In MAXMaps, we created conceptual maps displaying the targeted concepts, the interplay of their subthemes and the relationships among the concepts.

### Quotations

This study is a cross-language study. For example, based on a theory published in English, the data collection, analysis and interpretation were conducted in German, and the study results were published in English. There is a lot of methodological debate with regard to the meaning of quotations in qualitative research, particularly in a multilingual context [51–53]. In this study, we illustrated the reported results by including quotes from the 11 informal carer interviews. The first author (KK) selected the most relevant quotes, slightly smoothed out the language of the quotes in their German version and then translated them into English. To ensure ‘cross-language trustworthiness’ [54], the selection of quotes and the translation process were monitored by one co-author (CP), and the translation was reviewed by a professional editing service.

### Quality management

Throughout the research process, the following tools recommended by Kuckartz [49] were used to ensure methodological rigour: KK kept a research diary and performed extensive memorandum writing using the MAXQDA 2020 memo functions. Consensual coding was performed to evaluate the intercoder agreement. There were several consultations with the supervisor of K.K.’s doctoral thesis (M.R.) and with a colleague who has particular expertise in qualitative research methodologies (S.T.). To foster the validity of the developing results, periodic reflection meetings with the SoCA team were scheduled; furthermore, the results were discussed in a research colloquium with peer researchers at the DZNE Witten.

### Additional material

The questionnaire on sociodemographic data, the interview guide and the code system exist in the German language. This material will be provided on request by the corresponding author.

## Results

### Sample description

Data from *n* = 11 interviews with informal dementia carers who cared for 11 different persons living with dementia in 11 different dyads were analysed in this study. The sample included two wives and one husband who cared for their spouses and five daughters, one granddaughter and two sons who cared for one parent with dementia. A detailed sample description is provided in Table 1.

**Table 1:** Sociodemographic characteristics of the sample

**Informal carers** (***n*** **= 11)**	**Mean**	**Range**	**N** (%)
Age (general)	**57.0**	35–77	
* Age of caring spouses*	**76.0**	75–77	
* Age of caring children*	**49.9**	35–64	
Gender			
* Female*			**8** (72.7)
* Male*			**3** (27.3)
Professional occupation			**6** (54.5)
**Persons with dementia (*n*** **= 11)**	**Mean**	**Range**	**N (%)**
Age (of n = 6 PWD still alive)	**82.0**	74–89	
Gender			
* Female*			**8** (72.7)
* Male*			**3** (27.3)
**Dyads (n = 11)**	**Mean**	**Range**	**N (%)**
Kinship relation			
* Spousal relationship*			**3** (27.3)
* Parent–child relationship*			**8** (72.7)
Living situation			
* Same household*			**5** (45.4)
* Separated households in one house*			**2** (18.2)
* Separated households in same city*			**2** (18.2)
* Distance caregiving*			**2** (18.2)
Duration of the trajectory (in years and months)	**6y 3 m**	0y 8 m—15y 0 m	
Status of the trajectory			
* PWD living at home*			**4** (36.4)
* PWD institutionalised*			**2** (18.2)
* PWD deceased*			**5** (45.4)

Abbreviations: *PWD* person with dementia,*y* year, *m* month

### Dyadic relationship

**Fig. 3:**
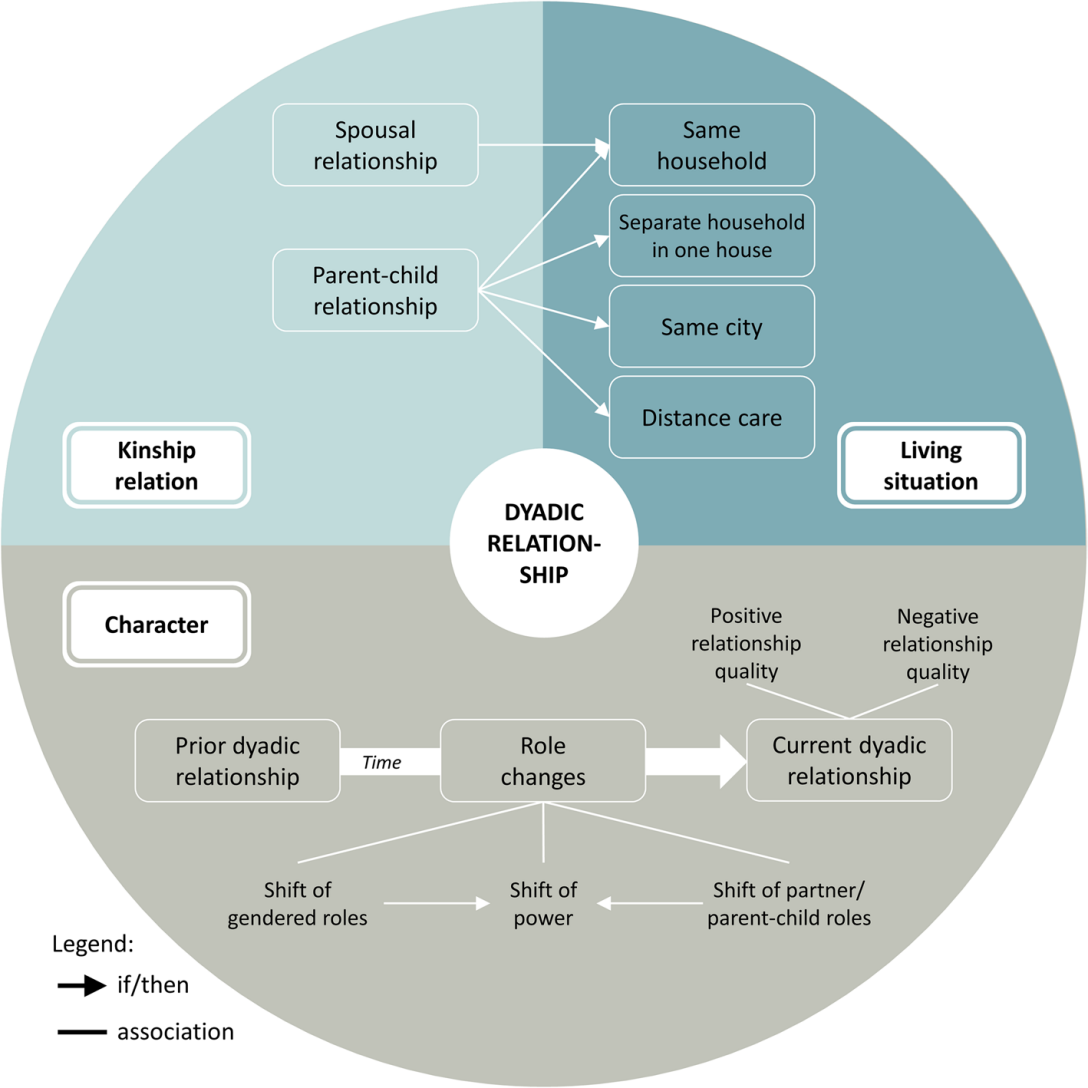
Visualisation of the concept dyadic relationship

The interviewees described their dyadic relationship along the themes of kinship relation, living situation and character of the dyadic relationship, which all comprise several subthemes. A visualisation of the concept and its themes and subthemes is provided in Fig. 3. In this paper, we provide the figures to serve as visual tools to support the reader to grasp at a glance the configurations of the concepts that are described in detail in the running text.

#### Kinship relation

Regarding the *kinship relation*, we distinguished between three spousal relationships and eight parent–child relationships. Four of the parent–child relationships were triadic rather than dyadic because the spouse of the person with dementia shared the care responsibility.

#### Living situation

Regarding the living situation, all of the spouses and two of the caring children lived in the same household as the person with dementia, two of the children lived in the same house as the person with dementia but had separate household, two of the children lived in the same city as the person with dementia, and two of the children provided care at distances of 300 and 900 kms. Accordingly, in the spousal relationships, there was maximum spatial proximity, and in the parent–child relationships, there was more diversity, ranging from close proximity to large distances.

#### Character of the dyadic relationship

The character of the dyadic relationship was shaped by a number of subthemes in a complex interplay. Subsequently, these subthemes are described individually.

The dyads entered the dementia care trajectory with a shared history in their prior dyadic relationship. Six informal carers provided insights into their prior dyadic relationship, as in their eyes, these had a remarkable influence on their current way of giving care. They reported decisive events from the biography of the person with dementia before the onset of dementia, talked about specific character traits and typical behaviour of the person with dementia prior to diagnosis as well as their own character traits, and described how these had shaped the character of the dyadic relationship in the past. For example, one caring son summarized how the conflict-ridden prior dyadic relationship affected his willingness to provide care for his mother:

*“Just sitting here and being almost always on call, day and night. After a short time, for me it was simply no longer bearable, although some people do this for years. One reason for this is certainly the overall strained relationship between mother and son, and everything that has happened over the decades.” EI-01.*

A strong topic related to the prior dyadic relationship that emerged in the analysis and interpretation process was the topic of 'power' within spousal relationships, parent–child relationships and families, which in turn was often associated with gendered roles. This topic was also sustained in the phase of role changes.

We found that with the onset and progression of dementia, the character of the dyadic relationship started to change. In eight interviews, shifts from long-standing roles were mentioned. Role changes became a core challenge for these dyads and shaped the dyadic relationship over a rather long period of time. Three typical role shifts were identified in the data: shift of gendered roles, shift of parent/partner-child roles, and, most prominently, shift of power. Three carers mentioned one role shift, and the others had experienced two or even all of them.

Regarding the shift of gendered roles, in the spousal relationships, the caring wives explained that they had taken on technical tasks or started to manage finances; conversely, the caring husband mentioned that he had taken responsibility for the housework formerly done by his wife. The new duties were experienced by the interviewees in two ways, sometimes as a burden but also as a positive challenge whose successful completion made the informal carer proud. For example, one caring wife repeatedly framed her dementia care journey as an emancipation process, and she noted:

*"I was the housewife, the mother of the children. And my job – I taught Dutch, and I made films for the West German Broadcasting Corporation and for local stations because I used to be a journalist in Holland – that still appealed a lot to me. But that was peanuts for him, he never really honoured that. And why am I telling this? Yes, well, the fifty-year marriage, the longstanding distribution of roles. Now it's the other way around." EI-03.*

Regarding the *s*hift of parent/partner-child roles, four caring children and one caring spouse clearly named the transition of the person with dementia as from being a parent or partner to being a child. Two daughters expressed that they had experienced this role shift (initially) as emotionally burdensome. Particularly, the caring children complained that their parent was no longer their role model or advisor but was in need of help. For the affected informal carers, this role shift was linked to the question of how one can successfully transition from the role of the cared-for child or from the role of the partner at eye level to the role of the supervising dementia carer. For example, one caring daughter stated:

*“Yes, you have to detach yourself from the scheme that you are a child yourself. But from then on you choose what you have to do to ensure that your mother is doing well. And that's incredibly difficult, because ultimately she's the person you respect, who always knew everything herself. And suddenly you can no longer take her serious as an adviser, because she simply doesn't know anymore.” EI-08.*

In particular, our findings highlight a *shift of power*, and thereby a change or even a reversal in leading and following in the dyadic relationship, as an important subtheme. Our analyses show that in the majority of the dyads, the question of who leads or follows was negotiated in dynamic processes throughout the prior dyadic relationship. With regard to relationship quality, how the dementia-related shift of power was managed and experienced by the two partners seemed to be crucial. According to the informal carers’ narratives, in the early phases of the trajectory, the people with dementia usually actively shaped their way of life and their care, whereas in late phases, the informal carers experienced them as increasingly passive and silent. For example, one husband described how actively his wife dealt with her diagnosis at the beginning of the trajectory, and how she communicated it in her social network:

*“And she knows everything, she also knows how it ends and dealt with it very intensively in the beginning. She still read the newspaper and also the books that we got about dementia. But she has dealt intensively with the diagnosis, also in the group discussions of the self-help group. […] And she's one of the very few—I mean, actually I don't know anyone who talks about it, yes? She says: Well, I have dementia. And most people don't even say that, do they? They can't get the word out of their mouths. And my wife is a person who says it.” EI-04.*

The informal carers perceived the active involvement of the person with dementia either as a welcome contribution to reciprocity in the dyadic relationship or – if the ideas of the person with dementia and their own ideas were not congruent – as a challenge. Hence, the informal carers reported that the shift of power was sometimes undesired or not accepted by at least one person, was in negotiation, or was clarified and accepted by both partners in the dyad. In the case of an undesired or not accepted shift of power, the informal carers stated that conflicts were daily occurrences and triggered feelings of burden. We found that the informal carers tended to achieve their goals either by carefully introducing the person with dementia to his or her desired outcome or by asserting themselves against the will of the person with dementia. For example, one caring son took over the official correspondence of his father, by promising that everything would further be done in his sense:

*„The situation has partly calmed down, with the paperwork and so. That's something that my father gave up willingly after a year or two. He is a very orderly person, and during the transition phase he always checked whether I was doing it as properly as he was. And I was able to show him very clearly that I was continuing his system. Then he was fine with it.” EI-02.*

In contrast, one daughter forced decisions without respecting her mother’s objection:

*“If she says I don't want that, I don't want to have a motion sensor in the bedroom […] My mother was against it. We don't have to talk about it. She perceived that as an absolute affront: I turn the light on! She didn't. Danger of tripping, in consequence, motion sensor.” EI-08.*

In the dyads that were in negotiation, this was framed by the informal carers either as arduous and burdensome or as a continuation of their previous form of relationship work and sometimes both. Finally, four informal carers noted that their daily life was quite easy because the shift of power had been clarified and accepted and the person with dementia appreciated the leadership of the informal carer. Our findings suggest that with the progression of dementia, the informal carers usually gained power. For example, one caring wife stated:

*“This is also a sign that he was no longer in control. Maybe he was happy too. He never said that, but maybe he was glad that someone was taking the initiative.” EI-03*

The shared history in the prior dyadic relationship and the processes of the role changes significantly shaped the character and quality of the current dyadic relationship. The majority of the informal carers described their dyadic relationship as a close and often loving one, and the subthemes of empathy, humour and togetherness appeared as prominent signals of the positive quality of the dyadic relationship. We found that in ten dyads, the informal carers acted empathically and tried to see the current situation through the eyes of the person with dementia. The empathic informal carers consciously observed the mood and the behaviour of the person with dementia, interpreted it in the context of the specific situation, and derived solutions from their empathic observations. These informal carers reported very positive experiences with this strategy. For one caring daughter, empathy was the core strategy through which to provide care:

*“I always ask her: How are you today? What did you do today? And then she says: I went to school today and I've already done my homework. Good, then I know where she is. […] So we talk about school or about classmates or about birds or where she is right now. Because that's her world and I join her.“ EI-07.*

The experiences of dyads that succeeded in keeping a sense of humour were also perceived as positive. In addition, togetherness was addressed as an indicator of the positive quality of the current dyadic relationship in seven interviews. The majority of informal carers found their daily life with the person with dementia worth living and valued the time shared. However, we found that the option to share pleasurable time together often decreased over time if the cognitive or physical limitations of the person with dementia increased. For example, one caring wife described her daily life:

*"My siblings say, why don't you move him to a care home, it's way too much for you? No, I will not do that. […] Because it works the way we do it. It's nice. I shave him in the morning and then I wait with him, read the newspaper with him a bit, until the nurse comes. And then I take him to day care, and Tuesday, when he's always at home, then I make appointments with the nail care, pedicure, then we have the doctor's appointments. […] And that's nice too. Yes, it is part of everyday life.” EI-03.*

In five dyads, a declining or negative relationship quality could be identified. Two relatives consciously limited the time they spent with the person with dementia or avoided contact at times, resulting in sharing increasingly less common ground. In approximately half of the interviews, the informal carers described (aggressive) conflicts and accusations that were made against the partner in the dyad. Both sometimes led to the emotional withdrawal of one of the partners. For example, one caring son complained:

*“I was even afraid to go downstairs. I was afraid to go downstairs and keep having this confrontation. Not because of her illness, but because of her behaviour. This aggressive behaviour, always aimed at me, always attacking me. Again and again, that was dramatic.” EI-01.*

If the informal carer consciously ignored the wishes of the person with dementia and enforced his or her own ideas, which was the case in six dyads, at least in phases, we interpreted this as another indication of the increasingly negative quality of the dyadic relationship. In our analyses, it became apparent that some informal carers provided examples that we interpreted as dominating the person with dementia to enforce their ideas and used or misused their power in this sense. This becomes visible for example, as one caring son reported:

*"I [the mother] don't need a cleaning lady, I'll still clean everything here. I'm still doing everything. Although, a cleaning lady came here for weeks, once a week, whenever she [the mother] was gone. She didn't even notice. So we practically organized the whole thing here behind her back." EI-01.*

### Carer role

**Fig. 4:**
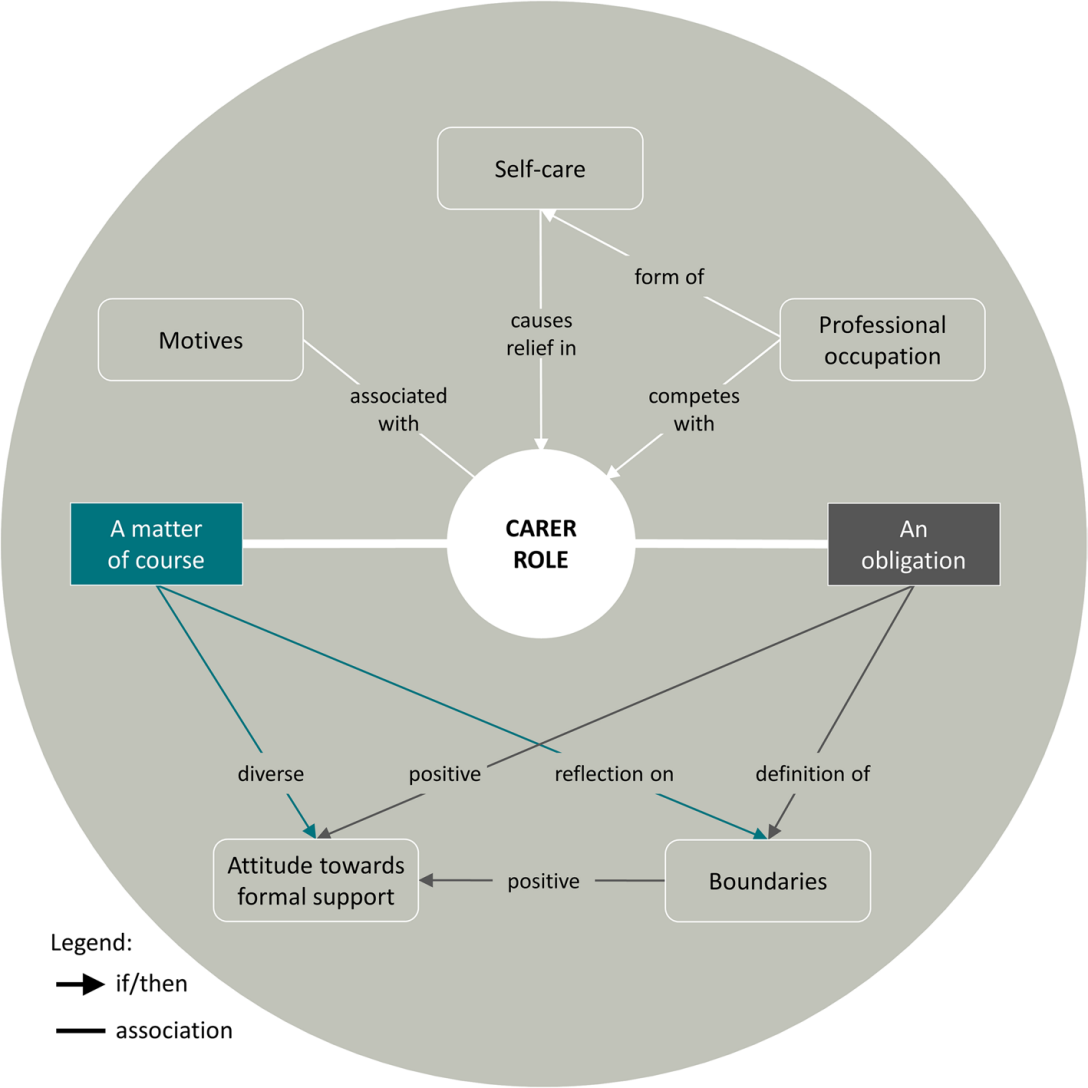
Visualisation of the concept carer role

The interviewees provided insights into their perception of the carer role. A visualisation of the concept and its subthemes is shown in Fig. 4.

All of the informal carers talked about the motives that led them to assume the carer role. The informal carers strove to keep the person living with dementia at home as long as possible and aimed to avoid nursing home placement. The motives of love, gratitude and reciprocity appeared in the narratives of five of the caring children. These children stated that they wanted to give back what they had previously received from their parents, and they wanted to make the rest of the care recipient’s or (if both parents were still alive) the older couple’s life as comfortable as possible. For example, one caring daughter stated:

*“That was important to me: my parents looked after us our life long, and they always tried to reach the best for us. And now it was my turn.” EI-07.*

The main finding concerning the carer role was that the basic attitude of the informal carer regarding his or her responsibility for a person with dementia strongly influenced their self-conception of their carer role, their individual experience, and their decisions and actions. We found that the majority of informal carers took the carer role *as a matter of course* because they interpreted it as a natural continuation of their dyadic relationship with the person with dementia. This applied particularly to spouses but also to caring children. In contrast, two informal carers experienced their carer role primarily *as an obligation*. Only rarely did these two forms occur in their pure form: usually, the feeling of a voluntary commitment was mixed (at least in phases) with the feeling of being obliged to care. However, we concluded from the informal carers’ narratives that which basic attitude (currently) predominated was decisive for the experience of the carer role. This mixture became visible as one caring daughter stated:

*“I already try to feel where I'm doing something out of obligation. […] A lot of things I may have done out of obligation at first, but it was actually fine to me. So that was just this sense of duty, it wasn't the real motivation. The real motivation was that I actually liked doing it. Out of love for my parents or for my mother.” EI-10.*

Informal carers who interpreted their carer role primarily or in parts as an obligation tended to define boundaries regarding their willingness to provide care, whereas informal carers who cared for their family member primarily as a matter of course addressed this issue only partially and with a different quality. In parent–child relationships, the caring children defined boundaries regarding their availability in the daily life of the person with dementia, specific practical care tasks, and the time they were willing to spend at the moment and in the future. The mentioned reasons for the definition of boundaries were either the desire to preserve a private life with a certain degree of freedom or the rejection of specific practical care tasks. For example, one caring son refused to assist his mother with physical care:

*“The most important support for me was that personal hygiene takes place in the morning. It was important for me that a nursing service came and provided this task. Because I can do all the household chores: shopping and making food and the whole works; I get everything solved. But this physical closeness and this mother-son relationship, I need support there. I can't manage that.” EI-01.*

In spousal relationships, boundaries were discussed more in the sense of expected physical or emotional exhaustion. For example, one caring wife explained:

*"I don't know how much longer I can do this. Sometimes you really think the limit is reached. […] The physical is more exhausting. The emotional, I've more or less come to terms with it. But this physical effort, always pulling him up, he doesn’t do anything anymore, he doesn’t wash himself, he doesn’t know when he needs to go to the toilet.” EI-05.*

Informal carers usually expressed a sense of burden if their defined boundaries were exceeded. Sometimes, however, contrary to expectations, as limits were reached, they did not present a problem; thus, they were constantly re-evaluated.

The interviewees had a diverse attitude towards formal support and the involvement of professional service providers in their care arrangements. Most importantly, the informal carers expressed a negative attitude towards nursing homes and the expected quality of care. This finding is associated with the most prominent motive for taking over the carer role: avoiding nursing home admission. Even when professional service providers were involved in all of the care arrangements, at least in phases, some informal carers argued that professional care could never be as beneficial as care provided by the family. For example, one caring daughter stated:

*"Everyone always advised us: put her in a nursing home, put her in a nursing home; but we didn't want to put her in a nursing home because we just felt that there was no nursing home in the world that could replace family. And that's still my conviction, that—when it's bearable within the family—it's the best way. That's a requirement, of course. But then no nursing home in the world can replace that, it doesn't work, that's nonsense." EI-09.*

The informal carers who argued in that way were often burdened by the assumption that they absolutely needed to persevere in their carer role, regardless of their exhaustion. Approximately half of the informal carers, those who partly felt obligated to provide care and those who defined their boundaries – for example, those who rejected assisting the person with dementia in intimate care tasks – appreciated professional support. In particular, 24-h live-in care was rated very positively. This was also the case for informal carers, who were rather reluctant to involve formal service providers. For example, one caring daughter shared her feeling of relief, after she had hired a live-in carer:

*“From the summer Misses [name] from Hungary was there. My mother was born in Hungary, so we thought it would actually be nice […] if someone spoke Hungarian to her too. And then they got along very well. And from then on a relief was noticeable. In all everyday life.” EI-10.*

Another aspect that became apparent in relation to the carer role in our analyses was professional occupation. Six interviewees – all of whom were caring children – were employed. Three carers who worked full-time and one carer who worked part-time perceived their work as competing with their caring duties. The informal carers who saw conflicting interests between work and care struggled to reconcile the two tasks and experienced this conflict as burdensome. For example, one caring daughter described:

*“Then I got her out of bed in the morning, made her breakfast, washed her, dressed her, got her ready for the day. Then I went to work. And in the evening, when I came home, I first went to my parents' house and looked after my mother and prepared dinner […] and then got her ready for bed again, washed her and so. And that worked for a relatively long time, until September, when I couldn’t bear it anymore, and then at some point I was at the end of my rope, so I hired a nursing service.” EI-09.*

However, in one interview, the carer’s professional occupation was described as a beneficial counterweight to caring and was therefore framed as a form of self-care.

Finally, eight interviewees mentioned self-care as a relevant aspect of their carer role. Common self-care strategies were the involvement of professional service providers and informal supporters in the care arrangement, taking time off and taking vacations, visiting self-help groups, and maintaining friendships, social contacts outside the family, and hobbies. For example, one caring wife explained, that she enjoyed when her husband visited the day care centre:

*“Yes, and the time that he isn’t here now, I don’t use it to turn the household inside out, I indulge in my hobbies. And that already helps." EI-05.*

The reduction of challenging behaviour was also framed as important in the context of self-care. In two cases, restricting or avoiding contact with the person with dementia was another self-care strategy. The interviewees framed self-care as a counterbalance to the burden of caring and therefore as a relief in their demanding carer role.

### Resources

**Fig. 5:**
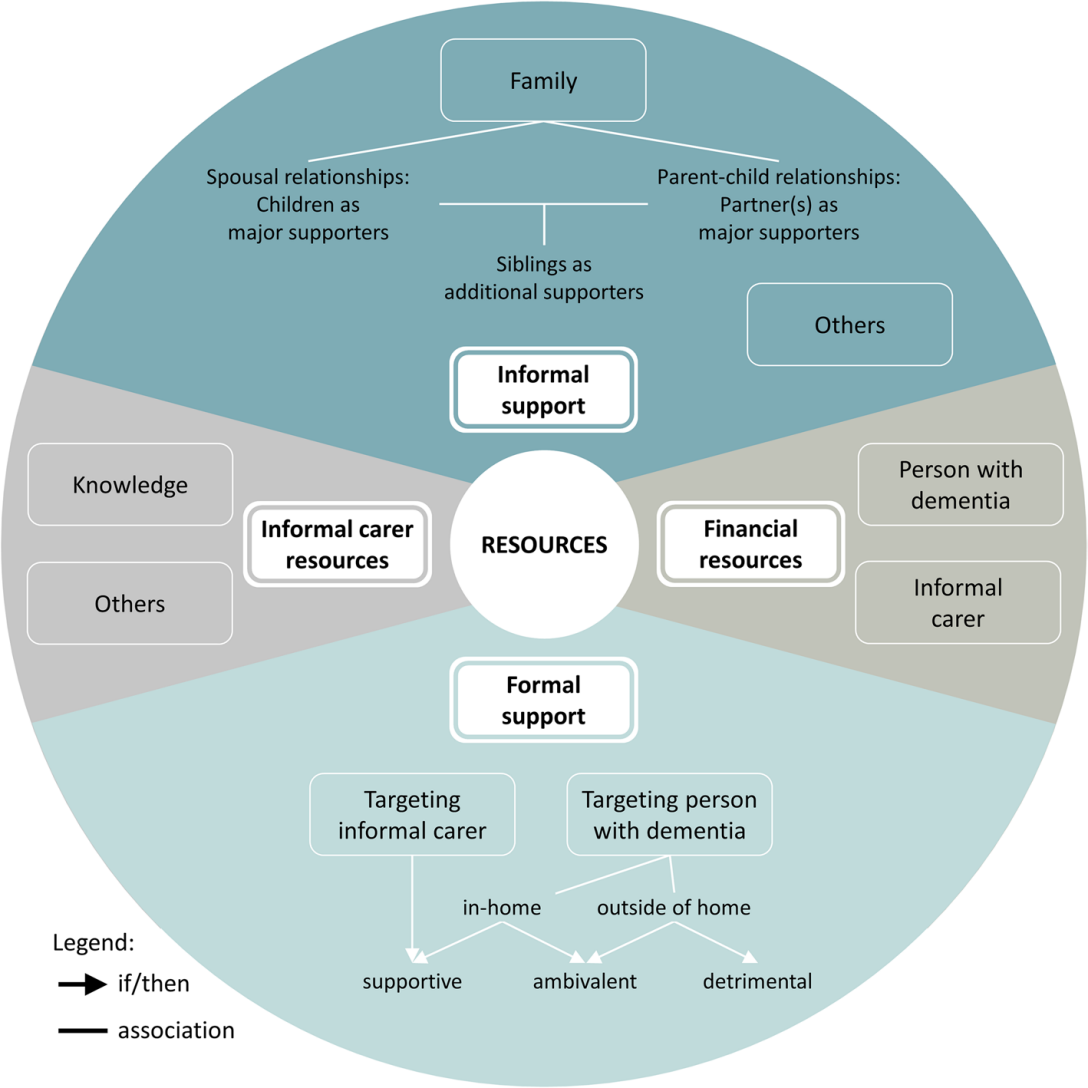
Visualisation of the concept resources

The interviewees’ statements in relation to the resources of their care arrangement could be grouped into four themes: personal resources, financial resources, informal support and formal support, all of which comprised various subthemes. A visualisation of the concept is shown in Fig. 5.

#### Informal carer resources

Regarding informal carer resources, the interviewees mentioned several character traits and skills that they perceived to be supportive in caring for a person with dementia. Humour, serenity, pragmatism, patience, organisational talent, a positive attitude towards challenges and being able to enjoy were mentioned casually in a few interviews. In contrast, nine of the informal carers highlighted *knowledge*. They pointed out the importance of gaining knowledge about dementia and its course, medical treatment, the availability and financing of formal support, and daily life with a person with dementia. For example, one caring granddaughter stated:

*“We dealt with the disease a lot. I have to admit that at the time I had absolutely no idea what dementia was. I was then at the university clinic and attended seminars to get information. I was lucky, suitable courses were offered at the time. My son then stayed with her. I then completed the seminars. Then I absorbed an unbelievable amount of literature, researched the internet, and then I had my own concept ready for how to deal with it. And from then on things went very well.“ EI-06.*

Two informal carers explicitly noted critically that it was difficult for them to receive relevant knowledge in a structured way. They experienced the fact that much helpful information reached them only by chance as a burden. Usually, the informal carers began to extend their knowledge immediately after the diagnosis and continued to learn throughout the entire care trajectory.

#### Financial resources

None of the interviewees in this study complained about problems due to a lack of financial resources. We found that frequently, a transfer of money had taken place across generations: caring children paid for formal support services for their parents who could not afford them. The fact that they had sufficient money was valued by the informal carers as a relief. While some informal carers used their financial resources straightforwardly as necessary, others (mostly children) reported that they had made strategic efforts to clarify what financial support from health and long-term care insurance they were entitled to so that they could use these funding opportunities as efficiently as possible. For example, one caring son stated:

*“She [the counsellor] knew exactly that I needed support from the nursing service here, and if it's just care level 0, this dementia care level. If only because it is partly financed if you give her to a day care centre or so. They do their billing related to entitlement by the law. Alone, so as not to just let the money go.” EI-01.*

#### Informal support

In this study, the informal carers valued informal support as a central resource for their care arrangements. By far the greatest importance was attributed to family members, whereas neighbours, friends and other informal supporters (e.g., concierges, volunteers, and church members) were marginally involved.

In all care arrangements, the informal carers valued their family members as a core resource. The more support the family members provided, the more the informal carers felt relieved in their carer role. If, on the other hand, the informal carer perceived the family support as inadequate, this triggered a noticeable burden. One caring daughter valued her dense family network:

*“And my sister, she lived in France, in Paris, came every four to six weeks. And my brother too. So we actually made a plan. That we children took turns. […] The other sister lived in Zurich, so it was far for her too, and my brother also worked abroad at the time. Nevertheless, they all came regularly. So there was like a duty-roster. Otherwise it wouldn't have worked. […] That was of course easy because we are four children. We distributed it well. And there were also grandchildren, who stepped in every now and then. This worked well.“ EI-10.*

In contrast, another caring daughter complained that her brother was not willing to support her:

*“I then asked him [the brother] for a few things when we had to deal with the insurance and stuff like that. I said come on, get the documents. Oh, I'm so far away, do it with someone on-site. Or it was about renovating the room for the live-in carer. And I said, can you take a Saturday off? Oh no, why, the live-in carer is on-site, she can help you too. You can do that together. So I felt very let down there. And that's why, as I said, I always had the main burden on my shoulders and sometimes I felt quite overwhelmed.” EI-07.*

We found that in the spousal care arrangements, one child of the couple was the most important supporter: this child either actively performed daily care tasks or was easily accessible as an advisor or conversation partner. In the parent–child care arrangements, the situation was more diverse. In the sample, there were care arrangements that benefited from a very large family network, including partners, siblings, children, grandchildren and other relatives, and there was one care arrangement in which a daughter cared for her mother in isolation. The informal carers reported that in the spousal and parent–child care arrangements, the supporting family members shaped the care arrangements through diverse functions: by providing practical care and emotional support, by serving as emergency backup, by supporting decisions, and by setting their own personal needs aside. Similar to the processes of role changes in the dyadic relationship, the interviewees also described the negotiations of roles between themselves and other involved family members. Therefore, the negotiation processes were sometimes associated with conflicts and experienced as burdensome. In other care arrangements, the care responsibility was negotiated between family members in dialogue and shared equally or divided according to individual capacities. For example, one caring daughter stated:

*“It has often been difficult because many sibling conflicts has broken out again. Who makes more? But that had strengthened us, this commitment, it was clear, that it should made possible for my parents to stay together. […] So it was actually a nice family dynamic, because of the binding character that this goal meant for everyone.” EI-10.*

*Friends* and acquaintances were mentioned in nine care arrangements. They acted as persons of trust who served as empathic conversation partners who could provide comfort and emotional backup. In one care arrangement, due to fear of stigmatisation, the husband with dementia and his wife had concealed the diagnosis in their circle of friends. In another case, the friends of the couple had withdrawn when the dementia became apparent. In none of the care arrangements were friends regularly involved in providing practical care to the person with dementia.

In six care arrangements, *neighbours* were mentioned as an informal resource. The neighbours lived close to the person with dementia: they kept an eye on what was happening next door, could be called in the case of an emergency, and occasionally dealt with a critical situation until the informal carer was on the spot. For example, without giving information to the caring daughter, one women living with dementia was discharged from the hospital and sent home by taxi. The daughter stated:

*“Then we were very lucky that the neighbour was at home, who also had a key from my parents. The taxi driver had simply disembarked her.” EI-07*

#### Formal support

We found that all of the care arrangements used formal support services and were in regular contact with health and social care professionals. Regarding support services that primarily target informal carers (e.g., counselling centres and self-help groups), the interviewees had mostly had very positive experiences and rated them as helpful in terms of gaining both practical information and emotional relief. For example, one caring son reports:

*“And that was kind of like a saving anchor for me. When I then experienced who knows what at the weekend, I called the [counsellor] on Monday morning: can I step by, I need help again. […] And then she got help somehow, in some way, even if she only talked to me heartening. Yes, sometimes that helps." EI-01.*

The experiences with support services that primarily target the person with dementia differed. The informal carers reported mostly positive experiences with in-home services (e.g., 24-h live-in care, visiting services, home help, and hospice services). With regard to ambulatory care services, the experiences were mixed: sometimes the use of an ambulatory care service was a great relief, and sometimes the informal carers criticised the organisation of the visits, the quality of care or the quality of contact with the nursing staff. For example, one caring wife praised the nursing service as very supportive:

*“I now have a nursing service in the morning and in the evening and that calms me down a lot, it relieves me a lot. And the aggressiveness between us has also subsided […]. I don't do it as nicely as the nurses do. And the nurses, I have to say they are very nice, they are very nice and patient, and they are also patient with him.” EI-03.*

In contrast, a caring granddaughter reported her negative experiences:

*“I had a notary appointment in another federal state. I had to go to my former hometown, therefore I was forced to hire a nursing service. And when I got home I was disgusted. Grandma had had diarrhoea that day of all days. She was just left in there and because it smelled very bad, the nurse spread several blankets over her to, yes, stop the smell, instead of washing and caring for her.” EI-06.*

Regarding formal support services outside the home, they told of both positive and negative experiences with general practitioners and specialists, day care centres and short-term care. Nine interviewees referred to contacts with the personnel of the health or long-term care insurance company of the person with dementia and also described mixed experiences: while some health insurance companies provided cooperative support, the negotiation of entitlements and financing of services with others were described as difficult. For example, one caring daughter stated:

*“And then being discharged home, this organizing. I didn't think it was possible; how difficult it is to even know what support is available. And then, how should I put it? I often felt like a petitioner. Or as if my request was almost an outrageous demand.” EI-10.*

In nine interviews, overly negative experiences were reported with regard to hospital stays of the person living with dementia as well as rehabilitation, which represented a great burden for the dyad. One caring daughter described:

*“I said in the hospital that I don't like leaving her there, because she's afraid when she's alone in strange surroundings, because one couldn't explain it to her anymore, and I asked them to stop giving her medicine. And if she actually did have screaming attacks again, I would be there in 20 min, I would also stay with her the night so that the nursing staff would not be burdened. She had this phase. But I wasn't called. I got there and my grandma was a zombie, all drugged up.” EI-06.*

To simplify, one could state that formal support services coming to the home of the person with dementia were predominantly experienced as a relief, while experiences with out-of-home formal services were mixed, and hospital stays were described as very burdensome.

In summary, this SDA provided insights into informal carers’ perceptions of selected concepts drawn from SoCA-Dem middle-range theory: the dyadic relationship, the carer role, and the resources of home-based care arrangements. The analysis enabled us to identify the central themes and subthemes within the researched concepts and to structure them. Therefore, this SDA addressed our first research question, and contributed to further clarification of conceptual vagueness within the SoCA-Dem meta-study.

### Interrelations between the concepts and their subthemes

In addition, this study aimed to analyse the interrelations between the three concepts of dyadic relationship, carer role, and resources.

#### Interrelations between dyadic relationship and carer role

Our findings revealed strong and multifaceted interrelations between the two concepts dyadic relationship and carer role. In all of the care arrangements included in this SDA, it became apparent that the informal carer’s experience of his or her dyadic relationship had a major influence on his or her self-conception and forming of the carer role and vice versa. Both concepts developed over time and in close interdependence: while the dyadic relationship was exposed to transition due to dementia, the informal carer simultaneously coped with the transition of becoming a family carer and with the adaptation to and development of that new role. The majority of informal carers described their dyadic relationship with the person with dementia as close, which was a strong motive for assuming the carer role. Informal carers who valued the character and quality of their prior and current dyadic relationship said that they mainly provided care as a matter of course. In contrast, informal carers who complained of a troubled prior or current relationship experienced the carer role (at least partly) as an obligation. Conversely, informal carers who provided care as a matter of course mostly described themselves as empathic and fostered reciprocal cooperation in their dyadic relationship. In contrast, we concluded from the narratives that informal carers who felt particularly obliged to care forced the shift of power, tended to ignore the wishes of the person with dementia and rather pushed forward their own ideas on how to manage that person’s life and care. In addition to this dynamic interplay between the character of the dyadic relationship and the forming of the carer role, our results indicate remarkable differences in the conceptual interrelation in accordance with the kinship relation of the informal carer and the person living with dementia. In spousal relationships, there seemed to be no need to explain the assumption of the carer role, whereas caring children shared arguments and justifications for why they had assumed the carer role and how they had formed it. In addition, caring children had often become the main carer simply because of their living situation: they were the person in the family who lived closest to the parent with dementia. We also observed associations between the kinship relation or living situation and the informal carer’s professional occupation, the defined boundaries and the informal carer’s attitude towards formal support: caring spouses were retired, whereas six caring children were employed and felt that their work was competing with their caring duties. Caring spouses lived with the person with dementia, whereas children who lived at a spatial distance logically experienced boundaries in their options to provide permanent supervision and direct care and consequently usually had a more positive attitude towards formal support.

#### Interrelations between carer role and resources

We also revealed interrelations between the informal carer’s self-conception and forming of the carer role and the denial or integration of *resources* in the care arrangements. Conversely, we analysed how available or unavailable resources as well as experiences of resources impacted the carer role. The informal carers commented that they had acquired increasing knowledge about dementia and how to care for a person living with dementia over time. This growing knowledge influenced their self-conception and forming of the carer role over the trajectory. Uncertainties at the beginning of the trajectory were replaced by the feeling of having become an experienced informal carer. Regarding the usage of formal resources, we found that informal carers who experienced their carer role primarily as an obligation, who defined boundaries, and/or who had a positive attitude towards formal support engaged formal support services to perform care tasks that they were unable or did not like to provide themselves. In contrast, informal carers with a negative attitude towards formal support were hesitant: they either assumed that formal care could never be as beneficial for the person with dementia as care provided by themselves, or they had had negative experiences with formal support in the past. Regarding the interrelation between the carer role and informal support, all informal carers valued support provided by their family members and friends. The more informal support was provided – either practical support or emotional backup – the more the informal carer felt relieved from and valued in his or her carer role. If informal carers lacked support from their informal network, they expressed role overload. The existence of both adequate formal and informal support enabled the informal carers to satisfy their own need for timeout and self-care, which also led to a feeling of being able to recover from the carer role and of being able to continue providing care.

#### Interrelations between dyadic relationship and resources

We also identified direct interrelations between the concepts dyadic relationship and resources. Caring children who lived some spatial distance from the person with dementia often pragmatically engaged formal support services to substitute for the care that they could not provide themselves.

In summary, this SDA confirmed our assumption of dynamic interrelations between the three concepts drawn from SoCA-Dem middle-range theory. It further clarified associations between specific themes and subthemes of the analysed concepts and thereby contributed to answering our second research question.

## Discussion

In this SDA, we analysed the informal carers’ perceptions of their dyadic relationship with a person living with dementia, of their carer role, and of the integration of resources into the home-based care arrangements. Therefore, we distinguished and linked the subthemes of the three concepts and revealed interrelations between the concepts and specific themes and subthemes. The kinship relation and living situation as well as the character of the prior and current dyadic relationship shaped the informal carers’ self-conception and forming of the carer role. This in turn influenced the integration of informal and formal resources into the care arrangement. Conversely, the amount and experienced quality of formal and informal support resulted in a feeling of relief or overload in the carer role. This again influenced the informal carer's way of living his or her current dyadic relationship with the person with dementia. The dyadic relationship, carer role and resources develop over the trajectory of dementia care and are exposed to constant change, and in their dynamic interplay, they also impact the stability of the care arrangements. Subsequently, we discuss our findings in relation to current research and to SoCA-Dem theory [38], which provided the theoretical frame for this study.

In this SDA, the dyadic relationship crystallised as the kernel of the care arrangement that fundamentally determines the dyad’s experience of living with dementia. This finding is mirrored by a multitude of elaborated reviews [5, 55, 56], syntheses [3, 57] theorisations [6, 58] and doctoral theses [59–61] that are dedicated to examining dyadic relationship trajectories in the context of dementia. The majority of these publications confirm our notion of a very close association between the dyadic relationship and the informal carer’s self-conception and forming of the carer role. For example, Ablitt [58], Conway [56], Luichies [62], and Bödecker [59] and their colleagues highlighted that the quality of the usually long-standing dyadic relationship before the onset of dementia is the main determinant of a strong commitment to the carer role. These publications also indicate that this major association between the two concepts of dyadic relationship and carer role is linked to the continuity or discontinuity of providing informal dementia care at home: a positive quality of the prior and current dyadic relationship argues for continuity over time, whereas low well-being in the prior and current dyadic relationship often causes a burdensome experience of the carer role and an earlier termination of home-based care. The novelty of this SDA is that we can now interpret the interrelation of the concepts in the frame of SoCA-Dem middle-range theory. Regarding our findings related to the concepts dyadic relationship and carer role, which are mirrored in other current research, we saw clear empirical evidence for the dense connection between the two concepts, and we could confirm our theoretical assumptions regarding their relevance for the creation of stable home-based care arrangements.

A subtheme addressed very prominently in our study is role changes, particularly the shift of power between the two people in the dyad. Power has often been negotiated over decades in marital relationships, and it was also a relevant aspect in the biographies of the adult children and their ageing parents. Gaining or losing power and the way in which this process was negotiated influenced the quality of the current dyadic relationship, the experience of the carer role and (related to the SoCA-Dem theory) the stability of the home-based care arrangement. There is substantial research that reports role changes within dyads coping with dementia and that – among other aspects – engages with power shifts [3, 6, 56, 59, 61, 63]. The named publications highlight the mutual collaboration of dyads while they strive to preserve their relationship, but they likewise point out struggles with a changing distribution of power, power imbalances, and sometimes the increasing powerlessness of the person with dementia. Altogether, the range of feelings associated with power shifts are diverse: while some partners relish their increasing power, others feel overloaded by their duties [3, 6, 63]. Quinn and colleagues [28] described a continuum of being dependent versus independent from the partner in the prior dyadic relationship, and they indicated that in particular, dependent informal carers struggled to adapt to their new leading role. In part, our findings show another picture: we interviewed caring wives who had long lived as the apparently weaker partner in their dyad but who enjoyed their chance to become the leading partner during their spouse’s dementia. In line with our findings, other studies also discuss the shift of power in terms of the idea of masculine or feminine roles [3, 6, 60, 61, 63]. The balance of power in dyads and the emotional reactions to the shifting power distribution are important if we aim to further understand the functioning or malfunctioning and the stability of home-based care arrangements in future research and in practice. As we did not find a study that exclusively researched the impact of power shifts, we recommend this approach as a valuable contribution to subsequent research.

In our SDA, noticeable distinctions became apparent between spousal and parent–child dyads. How the dyadic relationship interacted with the other concepts was often associated with the generational affiliation of the informal carer. Assuming the carer role was a matter of course for all spouses, whereas children tended to question their role quite often. The latest research illustrated that the kinship relation (carer from the same versus carer from a younger generation) is one of the most important dimensions in distinguishing between different types of home-based care arrangements [45, 64], and critics have noted that there is an underrepresentation of research on dyads in addition to intimate relationships [65]. Indeed, numerous publications have not differentiated between the generational affiliation of informal carers [55, 66] or have focused only on spousal relationships [3, 63]. Current research has begun to fill the noticeable knowledge gap with regard to parent–child relationships and to examine specific challenges in the context of filial dementia care. In particular, adult children struggle to integrate competing roles: they used to be not only informal carers but also spouses or partners, parents, friends and employees [62, 67]. Caring has a normative connotation for them: their decision to adopt or reject the carer role is strongly influenced by personal and family values, feelings of moral obligation, and adherence to cultural and societal norms [62]. Furthermore, caring children often need to cope with the vivid dynamics in wider family networks [29, 68]. In this complex area of conflict, adult children often experience providing care as simultaneously burdensome and rewarding. Future research that carves out the specifics of spousal versus parent–child dyads would further deepen our understanding of how stability can be created and maintained in these diverse types of care arrangements and how they could best be supported in practice.

In the majority of care arrangements included in this study, informal carers valued emotional and practical support from family members, which was experienced as a strong relief in the challenging carer role. This finding is in line with the latest research, which extends beyond a narrow dyadic perspective and provides insights into complex family networks and family dynamics. Marcum and colleagues [69] found that only a minority of care arrangements rely on a single primary carer but that in a plurality of networks, care tasks are distributed among multiple actors. This was also the case in the study of Neubert and colleagues [30], who explored family interrelations and identified four network types on a continuum between an unequal distribution of responsibility that worsens family relationships and care as a joint project that leads to increased family functioning. Esandi and colleagues [29] added a longitudinal perspective and portrayed family dynamics and their changes over time (from closeness to conflict or conflict to closeness and the diverse variations in between). Relevant topics at the dyadic level – such as relationship quality and role renegotiation – are also important at the family level and contribute to the overall complexity of care arrangement functioning. These findings indicate that family dynamics have a noticeable influence on the stability of care arrangements [29, 30, 60]; specifically, well-functioning family networks tended to find meaning in the shared care experience and balance changes almost effortlessly. In contrast, family networks that reported conflicts experienced an imbalance of sharing responsibility, burden, and failure to adapt. Hence, these care arrangements were more likely to become unstable. In line with the latest research, this SDA emphasised the relevance of informal resources in the form of dedicated family networks for the stability of home-based dementia care arrangements. This finding may initiate a discussion process within the SoCA team to reflect on whether the comprehensive resources concept of SoCA-Dem theory should be divided in future advancements of the conceptual model, whether ‘family resources’ may stand as a concept of its own, or whether the concept dyadic relationship changes to a wider ‘family relationships’ concept. In any case, future research and professionals in practice should always take into account the relevance of family dynamics and their complex influence on home-based care arrangements.

## Limitations

This SDA is the first examination of the applicability of the SoCA-Dem theory and of selected theoretical concepts in a qualitative empirical study. The conclusions drawn from this study provide a valuable contribution to the future refinement of the recently developed middle-range theory. Of course, this study has limitations. The interviews used for this SDA were conducted approximately three years before the finalisation of SoCA-Dem theory. The research team’s conceptualisation of stability has evolved since then, and if we were leading the interviews today, we would ask more specifically about the theoretical concepts. In accordance with SoCA-Dem theory, this study focuses on the informal carer perspective. We do not question whether the person with dementia has a vivid influence on the forming of the home-based care arrangement [2, 56, 70], and we advocate exploring and adding the perspective of people living with dementia in future research projects. Aditionally, the SoCA-Dem theory should be extended to include diverse actors’ perspectives regarding stability, ideally through participatory research approaches [71, 72]. In many aspects, the sociodemographic characteristics of the informal carers, persons living with dementia and dyads are comparable to the profiles reported in large quantitative samples in Germany [73, 74] and in an international context [75]. There are deviations in this study compared to the literature, as the proportion of caring children was significantly higher and the proportion of caring spouses was significantly lower than those in the cited quantitative studies. Unexpectedly, none of the study participants reported a lack of financial resources. In fact, we did not examine the financial backgrounds of the dyads and might have had a selection bias with regard to the socioeconomic status of the interviewees and people with dementia. Our study participants lived in Germany, were embedded in the German health care system and shared that cultural context. Hence, our findings may not be universally generalizable. Finally, most of our study findings are in accordance with existing empirical research in the field. The additional value of this study is that numerous assumptions from the SoCA-Dem theory could be empirically confirmed, and desirable areas for future investigation have been derived. Indisputably, however, further research must be performed to finally overcome the constitutive characteristic of the SoCA-Dem theory and to prove the causality of associations.

## Conclusions

This study adds to a more nuanced understanding of three concepts drawn from SoCA-Dem middle-range theory [38] and the interrelations of these concepts. The structure and character of the *dyadic relationship* shape the informal carer’s experience of the carer role and determine the use of resources. Conversely, the availability and experience of informal and formal support either promote or hinder a positive experience of providing care, which again affects the quality of the dyadic relationship.

According to Roy [42], a circular relationship of theory to research to practice is particularly evident in middle-range theories. The use of theory provides feedback for further theory development [44] and enhances the explanatory power of the theory over time [43]. This SDA has made a valuable contribution to this goal: the results extend beyond the rather descriptive character of the quite recently developed middle-range theory and pave the way for a desired causal modelling of the complex phenomenon of stability, for example, better operationalising stability, possibly in the form of an assessment instrument [41, 43]. We expect that in future research, stability may serve as a meaningful outcome [76] that aligns with the increasing call to focus on measurable positive aspects in the care journey of people living with dementia and their families [4, 77].

To further enhance our knowledge of how stable home-based care arrangements are created, there is a need to explore all actors’ perspectives on the complex dynamics within dyads, families, and the entire support network. For future research, we suggest choosing longitudinal designs to more precisely follow up on the dynamics of change and balancing in all phases of dementia care trajectories. Finally, theory-based and empirically grounded knowledge with regard to stability needs to be implemented in practice. Innovative counselling concepts, care interventions and care structures should be developed to support people with dementia and their informal carers in living and caring in the place of their choice while preserving a preferably high quality of life.

## Data Availability

Due to data protection reasons, the data used during the current study are available on reasonable request only. For this purpose, please contact the data managers of the Deutsches Zentrum für Neurodegenerative Erkrankungen (DZNE), site Witten, Germany (data-management-witten@dzne.de).

## Abbreviations

DZNE: Deutsches Zentrum für Neurodegenerative Erkrankungen
SDA: Secondary data analysis
SoCA: Stability of home-based care arrangements
SoCA-Dem: Stability of home-based care arrangements for people living with dementia
